# circRNA circAF4 functions as an oncogene to regulate MLL-AF4 fusion protein expression and inhibit *MLL* leukemia progression

**DOI:** 10.1186/s13045-019-0800-z

**Published:** 2019-10-17

**Authors:** Wei Huang, Ke Fang, Tian-Qi Chen, Zhan-Cheng Zeng, Yu-Meng Sun, Cai Han, Lin-Yu Sun, Zhen-Hua Chen, Qian-Qian Yang, Qi Pan, Xue-Qun Luo, Wen-Tao Wang, Yue-Qin Chen

**Affiliations:** 1 MOE Key Laboratory of Gene Function and Regulation, State Key Laboratory for Biocontrol, School of Life Science, Sun Yat-sen University, Guangzhou, 510275 People’s Republic of China; 2 The First Affiliated Hospital of Sun Yat-sen University, Guangzhou, 510080 People’s Republic of China

**Keywords:** Circular RNA, MLL-AF4 fusion protein, Leukemia progression

## Abstract

**Background:**

Circular RNAs (circRNAs) represent a type of endogenous noncoding RNAs that are generated by back-splicing events and favor repetitive sequences. Recent studies have reported that cancer-associated chromosomal translocations could juxtapose distant complementary repetitive intronic sequences, resulting in the aberrant formation of circRNAs. However, among the reported fusion genes, only a small number of circRNAs were found to originate from fusion regions during gene translocation. We question if circRNAs could also originate from fusion partners during gene translocation.

**Methods:**

Firstly, we designed divergent primers for qRT-PCR to identify a circRNA circAF4 in *AF4* gene and investigated the expression pattern in different types of leukemia samples. Secondly, we designed two small interfering RNAs specially targeting the back-spliced junction point of circAF4 for functional studies. CCK8 cell proliferation and cell cycle assay were performed, and a NOD-SCID mouse model was used to investigate the contribution of circAF4 in leukemogenesis. Finally, luciferase reporter assay, AGO2 RNA immunoprecipitation (RIP), and RNA Fluorescent in Situ Hybridization (FISH) were performed to confirm the relationship of miR-128-3p, circAF4, and *MLL*-*AF4* expression.

**Results:**

We discovered a circRNA, named circAF4, originating from the *AF4* gene, a partner of the *MLL* fusion gene in *MLL*-*AF4* leukemia. We showed that circAF4 plays an oncogenic role in *MLL*-*AF4* leukemia and promotes leukemogenesis in vitro and in vivo. More importantly, knockdown of circAF4 increases the leukemic cell apoptosis rate in *MLL*-*AF4* leukemia cells, while no effect was observed in leukemia cells that do not carry the *MLL*-*AF4* translocation. Mechanically, circAF4 can act as a miR-128-3p sponge, thereby releasing its inhibition on *MLL*-*AF4* expression. We finally analyzed most of the *MLL* fusion genes loci and found that a number of circRNAs could originate from these partners, suggesting the potential roles of fusion gene partner-originating circRNAs (named as FP-circRNAs) in leukemia with chromosomal translocations.

**Conclusion:**

Our findings demonstrate that the abnormal elevated expression of circAF4 regulates the cell growth via the circAF4/miR-128-3p/MLL-AF4 axis, which could contribute to leukemogenesis, suggesting that circAF4 may be a novel therapeutic target of *MLL*-*AF4* leukemia.

## Introduction

Recurrent chromosomal translocations have been implicated in multiple tumor types and create fusion genes that can promote oncogenesis [1–4]. However, the effect of chromosomal translocations on noncoding RNA remains largely unknown. Recently, Guarnerio et al. [5] reported that cancer-associated chromosomal translocations could, in addition to creating coding fusion mRNAs, juxtapose distant complementary repetitive intronic sequences, resulting in the aberrant formation of a type of noncoding RNA (ncRNA), termed circular RNAs (circRNAs), which are generated by back-splicing events that favor repetitive sequences. This type of fusion circRNA (f-circRNA) was found to increase the cellular proliferation rate and provide tumor cells a survival advantage upon therapy treatment both in vitro and in vivo. Their findings indicated that chromosomal rearrangements can drive tumorigenesis via circRNAs, highlighting that f-circRNAs might serve as potential therapeutic targets in fusion gene-mediated cancer.

CircRNAs are newly discovered ncRNAs that play important roles in various biological processes [6–11], including the occurrence and development of cancers [12–20]. circRNAs are highly stable due to their circular forms that lack any open end, effectively resisting degradation induced by exonucleases. It has been reported that circRNAs preferentially localize to and function in the cytoplasm, where they can serve as microRNAs (miRNAs) sponges to affect translational processing or directly bind to proteins to regulate protein localization and function [21–24]. An example is that circRNA CDR1as harbors multiple conserved binding sites for miR-7 and regulates its downstream genes expression [25]. Another example is circRNA_100290, which is highly expressed in oral squamous cell carcinomas and regulates CDK6 expression via binding to miR-29 family members [26]. In leukemia, although circRNAs were found to originate from fusion regions during gene translocation, named f-circRNAs, only a small number of these circRNAs were reported [5, 27]. For instance, only one circRNA, named f-circM9, was reported to be produced from the fusion region of *MLL*-*AF9* gene in leukemia. However, in *MLL*-rearranged leukemia, the *MLL* gene can be fused with one of over 100 partners, including *AF4*, *AF9*, and *ENL*, to form more than 100 *MLL* fusion genes [28]. We question whether circRNAs could also originate from partners during *MLL* translocation and whether the formed circRNAs from the partners also have an effect on fusion genes and leukemic cell activities.

To address this question, we analyzed a circRNA profiling data set recently available [29]. This data set comprised a number of circRNAs from their parent genes including the *AF4* gene, a partner of the *MLL* fusion genes in *MLL*-*AF4* leukemia [30], which is predominantly found in acute lymphoblastic leukemia (ALL) [31]. We discovered a circRNA, named circAF4, which originates from the *AF4* gene loci. In this study, we showed that circAF4 plays an oncogenic role in *MLL*-*AF4* leukemia and promotes leukemogenesis in vivo. Mechanically, circAF4 can act as a miR-128-3p sponge, thereby releasing its inhibition on *MLL*-*AF4* expression. Knockdown of circAF4 increased the leukemic cells apoptosis rate in *MLL*-*AF4* cells, while no effect was observed in leukemia cells that do not carry the *MLL*-*AF4* translocation. This finding indicates that circAF4 may have biological mechanisms that are relevant to the tumorigenesis of fusion proteins, highlighting that this type of fusion gene partner-derived circRNAs (we named it as FP-circRNAs) may play important roles in fusion gene-mediated leukemia.

## Materials and methods

### Cell lines and cell cultures

The human leukemic cell line RS4;11, SUPB15, and MV4-11, and human embryonic kidney cell HEK-293T were cultured in RPMI-1640 (HyClone, USA) and DMEM (Gibco, USA), respectively, supplemented with 10% fetal bovine serum (Gibco, USA) at 37 °C in a 5% CO2 atmosphere.

### Patient samples

A total of 52 bone marrow samples including 22 *MLL* rearrangement leukemia samples, 30 non-*MLL* rearrangement leukemia samples taken from the time of diagnosis and 16 samples from healthy donors were included in this study. The patient characteristics are summarized in Table 1. All samples were obtained with informed consent from the first Affiliated Hospital of Sun Yat-sen University. Sample collection was approved by the Hospital’s Protection of Human Subjects Committee.

**Table 1:** Clinical characteristics of all samples used in the study

Type of sample	Characteristics	Median (range)	No. (%)
MLL-AF4 (*N* = 7)	Age at diagnosis, years	1 (0.25–7)	
	Sex		
	Male		4 (57.1)
	Female		3 (42.9)
	Immunophenotype		
	B		7 (100)
	T		0 (0)
	WBC count, × 10^9^/L	89 (18.29–173)	
	Risk group		
	H		4 (57.1)
	M		0 (0)
	S		0 (0)
	N/A		3 (42.9)
MLL-other fusion type (*N* = 15)	Age at diagnosis, years	4 (0.33–13)	
	Sex		
	Male		11 (73.4)
	Female		4 (26.6)
	Immunophenotype		
	B		10 (66.7)
	T		5 (33.3)
	Fusion gene		
	*MLL*-*AF9*		1 (6.7)
	*MLL*-*ENL*		2 (13.3)
	*MLL*-*AF10*		1 (6.7)
	N/A		11 (73.3)
	WBC count, × 10^9^/L	72 (4.63–370)	
	Risk group		
	H		9 (60)
	M		3 (20)
	S		1 (6.7)
	N/A		2 (13.3)
MLL-wt (*N* = 30)	Age at diagnosis, years	8 (1–13)	
	Sex		
	Male		19 (63.3)
	Female		11 (36.7)
	Immunophenotype		
	B		12 (40.0)
	T		12 (40.0)
	N/A		6 (20.0)
	WBC count, × 10^9^/L	31 (2–632.47)	
	Risk group		
	H		7 (23.3)
	M		12 (40)
	S		5 (16.7)
	N/A		6 (20)
Normal (*N* = 16)			16 (100)

### RNA extraction and qRT-PCR

The nuclear and cytoplasmic fractions were extracted using NE-PER Nuclear and Cytoplasmic Extraction Reagents (Thermo Scientific, USA). Total RNA from whole-cell lysates or the nuclear and cytoplasmic fractions were isolated using TRIzol (Life Technologies, USA). RNA was reverse transcribed to cDNA using PrimeScript™RT reagent Kit with gDNA Eraser (Takara, Japan). The expression level of AF4 and circAF4 were measured by quantitative PCR using SYBR Premix Ex Taq™ (Takara, Japan). The RT-PCR primers used were as follows (5′-3′): circAF4(EX3-4)-F: GCTCTCCAAAAAGGGGAATC, circAF4(EX3-4)-R: CCCCTG AACTGAAACCACTG, circAF4(EX3-5)-F: AGCCATCCAAGTTTCCTT TC, CircAF4(EX3-5)-R: TGGTTGCGTCTTTCCTTCTC, circAF4(EX5-6)-F: CAACATAGCCCACTGAAATA, circAF4(EX5-6)-R: TGAACTCACCTGGAAAGATA, circAF4(EX4-12)-F: TGTCAGTTCTGTAACCAA, circAF4(EX4-12)-R: GGAATTAAAGGATATTTCG, AF4-mRNA-F:AAACCACTGCCGGAGGACTAT, AF4-mRNA-R: GTATTGCTGTCAAAGGAGGCG. GAPDH-F: GAAGGTCGGAGTCAACGGATTTG, GAPDH-R: ATGGCATGGACTGTGGTCATGAG.

### RNase R treatment

RS4;11 and leukemia primary cell DNase-treated total RNA (5 mg) was incubated 20 min at 37 °C with or without 3 U μg^−1^ of RNase R (Epicenter Biotechnologies, USA). RNA was subsequently purified by GeneJET RNA Purification Kit (Thermo Scientific, USA).

### Oligonucleotide interference and plasmid construction

miRNA mimics and small interfering RNAs (siRNA) were synthesized by GenePharm (Shanghai, China) and Ribobio (Guangzhou, China), respectively. The 293T cells were transfected using Lipofectamine 2000 (LifeTechnologies, USA). The leukemia cell line RS4;11 and MV4;11 cells were electrotransfected by Neon™ Transfection System. The luciferase reporters with the inclusion of AF4 or circAF4 sequence containing miR-128-3p binding sites were constructed by cloning indicating fragment into psi-check2 vetor (Promega). The mutant reporters were constructed by mutating 3 nucleotides which were perfect complementarity to miR-128-3p. All constructs were verified by sequencing.

### Construction and transfection of circAF4 overexpression vector

The method used to construct circRNA overexpression vector was as described previously [32]. In brief, we used the introns from *SUZ12* to facilitate circRNA product. All intron sequences were cloned in pCDH-CMV-MCS-EF1-Puro-copGFP vector. Sequences of exon 3-4 of *AF4* gene were inserted into the vector. We named this vector for circAF4 overexpression as PCDH-circAF4. For stable overexpression of circAF4 in leukemia cell RS4;11, PCDH-circAF4 vector was packaged into lentiviruses using Lentivector Expression Systems (System Biosciences, Germany), consisting of pPACKH1-GAG, pPACKH1-REV, and pVSV-G vectors, which were co-transfected in 293T cells using the Lipofectamine 2000/3000 (Invitrogen, USA) system according to the manufacturer’s guidelines. Finally, the lentiviruses were transformed into RS4;11 cells, and the transformed cells were then selected with puromycin.

### Dual luciferase activity assay

293T cells were transfected with 20 nM miR-128-3p mimics or negative control; 50 ng of psiCHECK2 control or psiCHECK2-AF4-1, psiCHECK2-AF4-2, psiCHECK2-circAF4 or psi-CHECK2-point mutated vector. Firefly and Renilla luciferase activities were measured consecutively 24 h following transfection using the Dual-Luciferase Reporter Assay (Promega, USA) according to the manufacturer’s instructions.

### Western blot

Cells were washed twice with ice-cold phosphate-buffered saline (PBS) and ruptured with RIPA buffer (Beyotime, China) containing 10% PMSF. Cell lysates were resolved by SDS-PAGE and transferred onto PVDF membranes. Membranes were blocked for 1 h with 5% BSA and incubated overnight at 4 °C with anti-MLL antibody (#14689, Cell Signal Technology, USA), and anti-tublin antibody (Sigma, USA). Membranes were washed three times with TBST, incubated for 1 h with appropriate secondary antibodies conjugated to horseradish peroxidase, and developed using chemiluminescent substrates.

### Apoptosis analysis and cell proliferation assays

RS4;11 and MV4;11 cells were collected at 48 h after transfection. Apoptosis was assessed using Annexin V, FITC Apoptosis Detection Kit (AD10, Dojindo, Japan) following the corresponding manufacturer’s manuals. The proliferation of RS4;11 and SUPB15 cells was tested by CCK-8 kit (Dojindo, Japan). Approximately transfected 2.6 × 10^4^ cells in 100 μl were incubated in triplicate in 96-well plates. At 0, 24, 48, 72, and 96 h, the CCK-8 reagent (10 ml) was added to each well and incubated at 37 °C for 3 h. The cell proliferation was also tested by EdU assay using Cell-Light EdU Kit (RiboBio, China). RS4;11 cells were seeded in 48 wells, 48 h after electrotransfected with si-circAF4 or negative control (NC) oligonucleotide. Cells were added with 50 mM EdU and incubated for another 4 h. Cells were then fixed with 4% paraformaldehyde and stained with Apollo Dye Solution for proliferating cells. Nucleic acids in all cells were stained with Hoechst 33342. The cell proliferation rate was calculated according to the manufacturer’s instructions. For cell cycle analysis, transfected cells were harvested and washed twice with cold PBS, and the Cell Cycle Kit (MULTISCIENCES, Hangzhou, China) was used according to the manufacturer’s guidelines. The detection was performed with a FACS Calibur using CellQuest software (BDIS, San Jose, CA, USA).

### RNA FISH

In situ hybridization was performed using specific probes to circAF4 sequence purchased from Ribobio (Guangzhou, China). Hybridizations were performed according to the manufacturer’s instructions of Fluorescent in Situ Hybridization Kit (Ribobio, Guangzhou, China).

### RNA immunoprecipitation

RNA immunoprecipitation (RIP) experiments were performed by using the Magna RIP RNA-Binding Protein Immunoprecipitation Kit (Millipore, Bedford, MA) according to the manufacturer’s instructions. Approximately 1 × 10^7^ cells were pelleted and re-suspended with an equal pellet volume of RIP Lysis Buffer (about 100 μl) plus protease and RNase inhibitors. The cell lysates were incubated with 5 mg of control mouse IgG or antibody against AGO2 peptide (Sigma-Aldrich) coated beads with rotation at 4 °C overnight, respectively. After treating with proteinase K, the immunoprecipitated RNAs were extracted by phenol-chloroform extraction.

### Animal model

Five-week-old male NOD-SCID mice were maintained under specific pathogen-free conditions in the Laboratory Animal Center of Sun Yat-sen University. All experimental procedures were performed according to the institutional ethical guidelines for animal experiments. Direct injection of 5 × 10^6^ short hairpin RNA (shRNA) transformed RS4-11 cells in 150 μL of PBS was performed to establish intravenous (tail vein) leukemia [33]. For the control, 150 μL of PBS without cells was injected. Three mice in each group were sacrificed after 2 weeks. Subsequently, the blood and organs from xenograft mice were treated with a red blood cell lysis buffer (Biolegend, USA), and cells washed with PBS containing 2% FBS before cell cytometry. Flow cytometry for the GFP+ % of transduced RS4-11 cells was performed on a BD FACScalibur cytometer (BD, USA) and analyzed using FlowJo software. Five mice left were performed the survival assay.

### Statistical analysis

Unpaired *t* test was used for two-group comparison, and data are expressed as the mean ± SEM of three independent experiments. The compassion among three or multiple groups was performed by one-way ANOVA, and the Tukey’s Multiple Comparison Test was used to analyze multiple comparisons. The probability of leukemia-free survival at 5 years was the study end-point. Leukemia-free survival was calculated from the date of complete remission (CR) until either relapse or death in remission [33]. We used the Kaplan-Meier method with a log-rank test to analyze the leukemia-free survival. Two-tailed tests were used for univariate comparisons. *p* < 0.05 was considered statistically significant.

## Results

### Identification and characterization of circAF4 in leukemia with *MLL*-*AF4* translocation

**Fig. 1:**
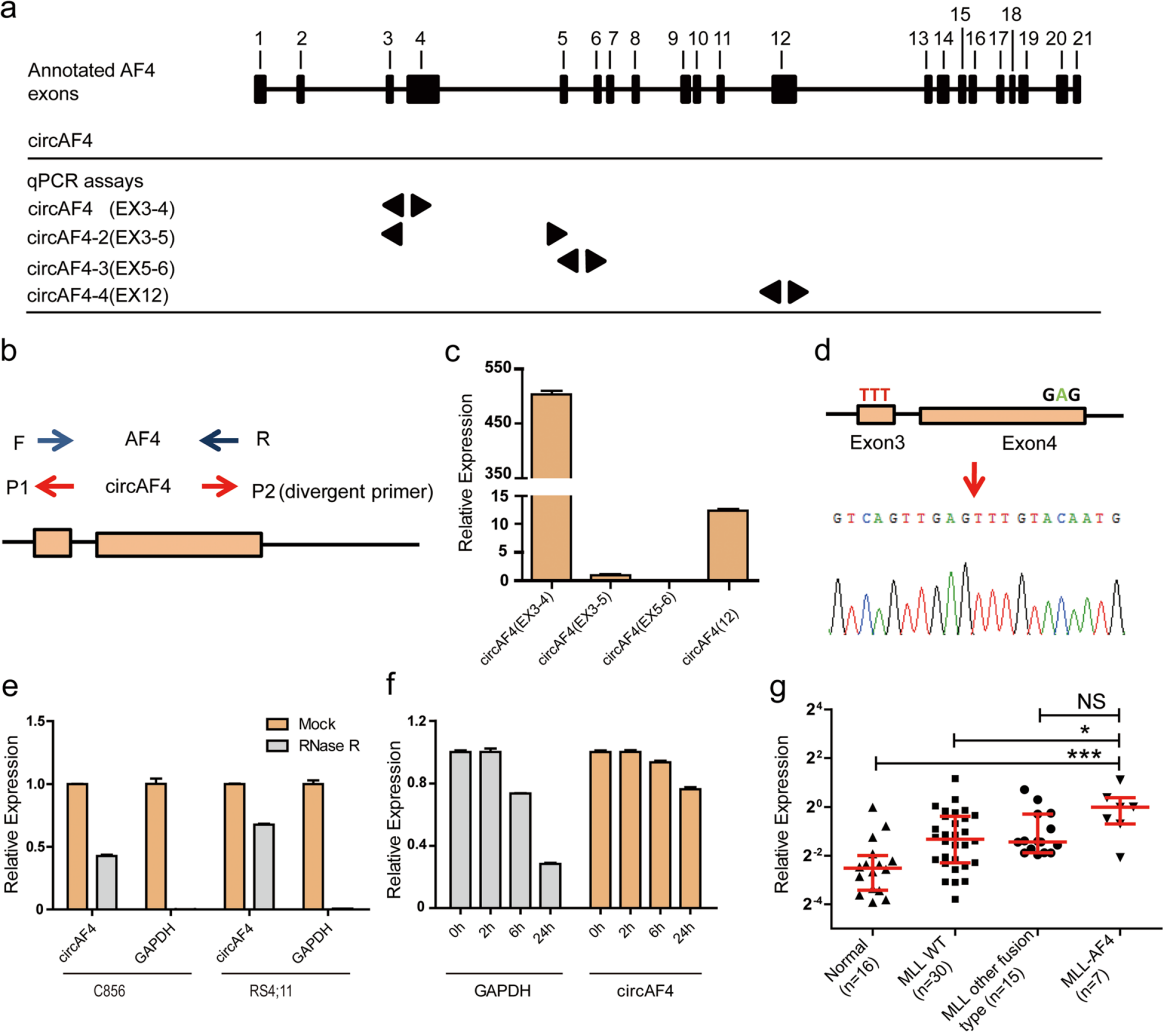
Characterization of circAF4 in leukemia cells. **a** Schematic of the genomic loci of four circRNAs in *AF4* gene. Arrows represent the length of indicated circRNAs. **b** Divergent primers were used for circRNA validation. **c** RNA was isolated from human leukemia cell line, RS4;11. The expressions of four circAF4 isoforms were validated by qTR-PCR. Expression was calculated using the 2^−ΔΔCT^ method and normalized to GAPDH. **d** The qTR-PCR product of circAF4 was validated by sanger sequencing. The arrow represents the unique junction site of circAF4. **e** qRT-PCR for the abundance of circAF4 and GAPDH mRNA in cells treated with RNase R. The amount of circAF4 and GAPDH mRNA were normalized to the value measured in the mock treatment. **f** Transcription was blocked by adding 2 μg ml^−1^ actinomycin D to the RS4;11 cell culture medium. RNAs were extracted in the indicated time points. The qTR-PCR analysis was performed as described above. NS, not significant. *P < 0.05, ***P < 0.001. **g** The qTR-PCR analysis of the relative expression of circAF4 in leukemia patients and healthy people. NS, not significant. *P < 0.05, ***P < 0.001

To characterize circRNAs derived from *AF4* gene, we systematically investigated the exon structure of *AF4* in the human genome. Four circRNA isoforms were identified originating from different exons of *AF4* gene in previous circular RNA profiling study [29]. We termed them as circAF4(EX3-4), circAF4(EX3-5), circAF4(EX3-4) and circAF4(EX12), respectively (Fig. 1a). To demonstrate the existence of endogenous circAF4s in leukemic cells, we used divergent primers (Fig. 1b) and qRT-PCR to amplify the four cirRNAs isoforms in RS4;11 cells, a cell line carrying the *MLL*-*AF4* translocation [34]. We noted that one of these four circRNA isoforms, termed circAF4(EX3-4) and derived from the *AF4* gene Exon3 and Exon4, was the predominant isoform with a very high expression level, approximately 550 and 40 times higher than circAF4(EX3-5) and circAF4(EX12), respectively (Fig. 1c). CircAF4(EX5-6) was undetectable in our analysis. Thus, we mainly focused on circAF4(EX3-4) for following functional investigation and referred it as circAF4. The unique back-spliced junction point of circAF4 was validated by amplification with outward-facing primers and then sequencing (Fig. 1d). To verify that the back-spliced events were indicative of the true circular ones, and not linear, trans-splicing, or genomic rearrangement products, we examined the physical properties of circAF4. circRNAs are highly stable compared to linear counterparts [25]. We digested the RNA extracted from RS4;11 cells and the primary cells from a *MLL*-*AF4* leukemia patient C856 with RNase R [35], an exonuclease that only degrades linear RNA molecules. The linear housekeeping gene GAPDH mRNA was used as a control to indicate the activity of RNase R. As shown in Fig. 1e, the linear GAPDH mRNA was nearly eliminated after RNase R treatment, while the circular circAF4 was only slightly affected. To further confirm this observation, we treated RS4;11 with Actinomycin D [36], a drug could inhibit polymerase II transcription and blocks mRNAs and circRNAs biogenesis, to examine the degradation rate of circAF4 in vivo. We quantified the expression level of circAF4 and GAPDH at the indicated time points. Corresponding with the observation above, circAF4 turned over more slowly than GAPDH (Fig. 1f), suggesting that circAF4 was highly stable in cells. Thus, the results further confirm that circAF4 is a true circular molecular.

Since circAF4 derived from *MLL* fusion partner gene *AF4*, we then asked whether circAF4 was associated with *MLL*-*AF4* leukemia. We recruited a cohort of 52 leukemia patients, including 22 patients with different MLL fusion proteins and 30 patients without fusion proteins, and 16 normal people to detect circAF4 expression (the detailed clinical parameters are presented in Table 1). As shown in Fig. 1g, the expression level of circAF4 was significantly increased in *MLL*-*AF4* leukemia patients compared with leukemia patients without *MLL* rearrangement and healthy people, and also in the patients with other *MLL* fusions although the *p* value was not significant due to the small data set. These results suggested that circAF4 might play a specific role in *MLL*-*AF4* leukemia. Next, we evaluated the clinical value of the aberrantly expressed circAF4. When clustering patients into three risk stratification groups, standard risk (S), middle risk (M) and high risk (H), based on the risk stratification system from ALLIC BFM 2002, we found that the patients in the high risk group had a highest circAF4 expression, showing progressive changes of cricAF4 expression along with the severity of the disease (Additional file 1: Figure S1a). The results also showed that patients who have higher white blood cells (WBC) may express higher levels of circAF4 (Additional file 1: Figure S1b). These results suggested that circAF4 may have a function in the aggressive leukemia.

### CircAF4 plays an oncogenic role in *MLL*-*AF4* leukemia

**Fig. 2:**
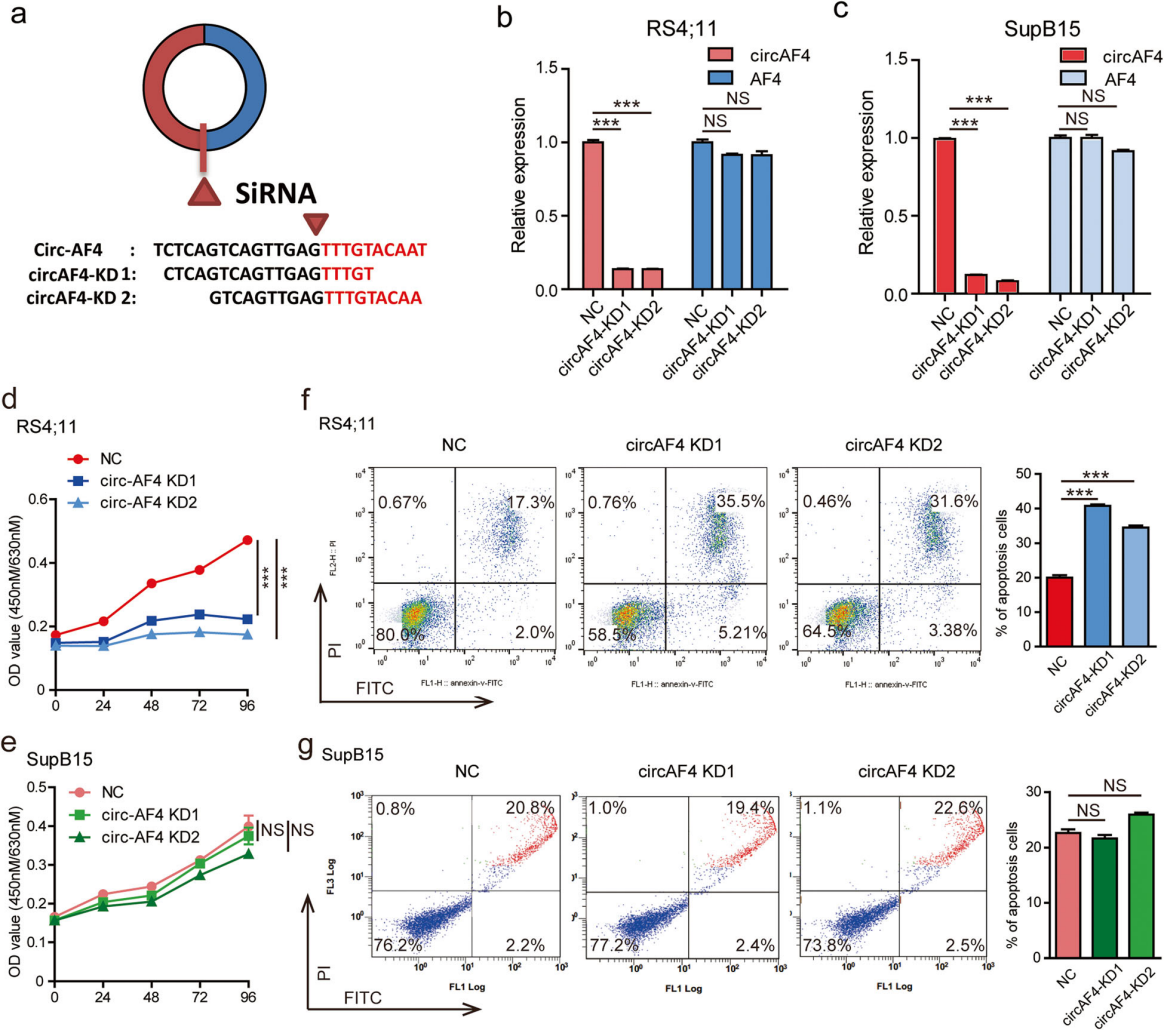
CircAF4 plays an oncogenic role in *MLL*-*AF4* leukemia in vitro. **a** Schematic illustration showing the junction site of circAF4 and two targeted siRNAs. qRT-PCR for circAF4 and AF4 mRNA in RS4;11 (**b**) and SupB15 (**c**) cells treated with two siRNAs as described above. NS, no significant. ***P < 0.001. Proliferation of RS4;11 (**d**) and SupB15 (**e**) cells transfected with the above two siRNAs assessed using a CCK-8 kit (Dojindo, Japan) at the indicated days. NS, no significant. ***P < 0.001. RS4;11 (**f**) and SupB15 (**g**) cells were exposed to 10 μM and 3 μM dexamethasone, respectively, for 18 h before collection at 48 h after transfection. A representative experiment is shown. NS, no significant. ***P < 0.001

Next, we investigated whether a higher level of circAF4 is specifically required to sustain proliferation and inhibit cell death in the context of *MLL*-*AF4* rearrangement. Two B-cell ALL cell lines, RS4;11 and SupB15, with or without *MLL*-*AF4* fusion gene, respectively, were used for the following studies. We used two small interfering RNAs (si-circAF4) specially targeting the back-spliced junction point of circAF4 as shown in Fig. 2a to reduce circAF4 expression levels. A nonspecific siRNA sequence was employed as a control. The specificity of the siRNAs directly against the backsplice sequence of the circular transcript was observed; these two siRNAs only knocked down the circular transcript and did not affect the expression of linear species (Fig. 2b and c). We next performed CCK8 cell proliferation assay. As shown in Fig. 2d, downregulation of circAF4 significantly decreased the growth of RS4;11 cells, but had limited effects on SupB15 cells (Fig. 2e). An EdU incorporation assay and cell cycle assay further confirmed that the cell proliferation of RS4;11 cells was impaired when interfering circAF4 expression (Additional file 1: Figure S1c and d). In addition, we measured the apoptosis rate by knockdown of circAF4, and the results showed that apoptosis was induced in RS4;11 cells (Fig. 2f), while no effect was observed in SupB15 cells (Fig. 2g). Together, it can be implied that circAF4 functions as an oncogene in leukemia with *MLL*-*AF4* translocation.

### CircAF4 promotes leukemogenesis in vivo

**Fig. 3:**
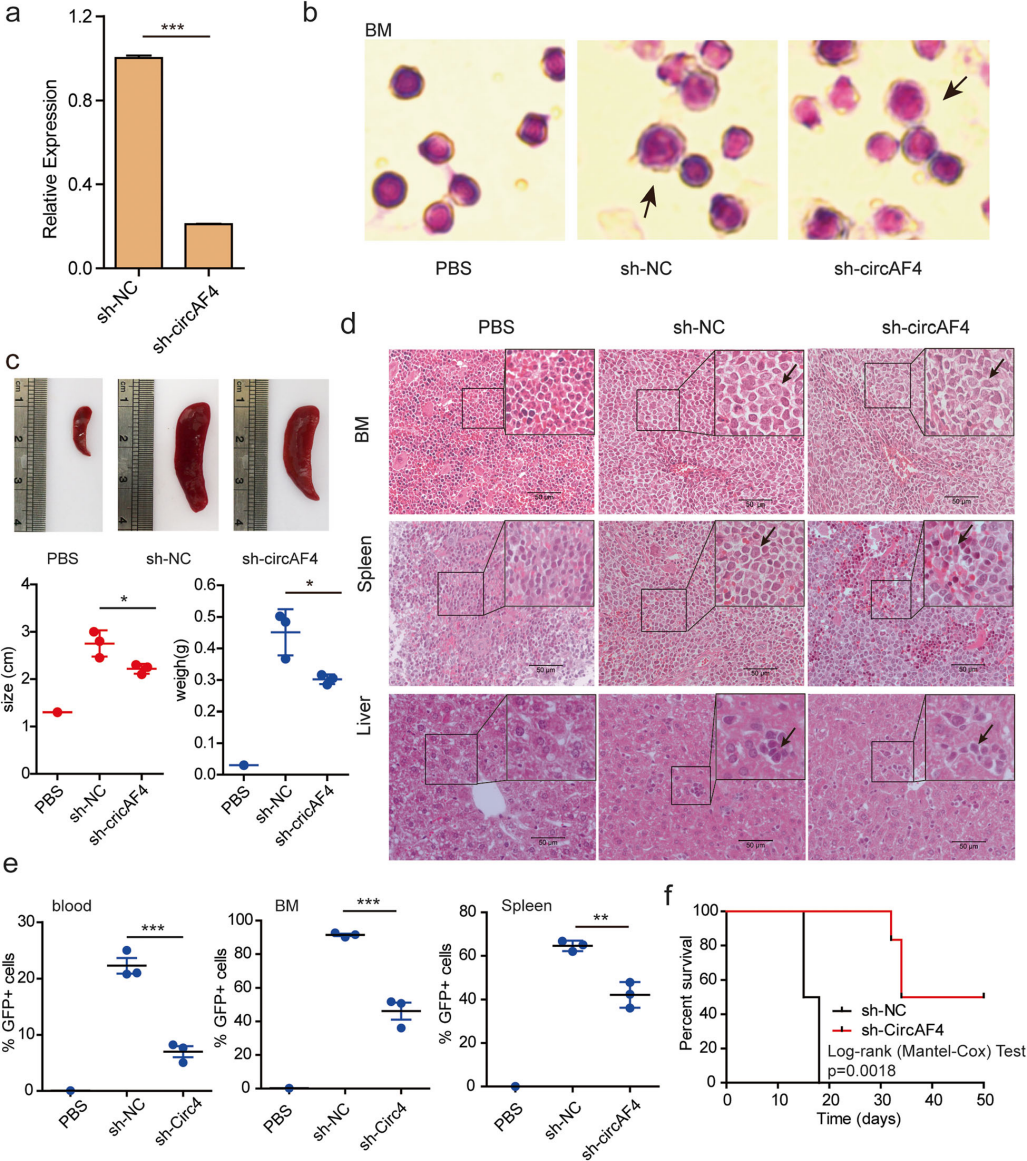
CircAF4 promotes leukemogenesis in vivo. **a** qRT-PCR confirmation of knockdown of circAF4 by lentiviral constructs in RS4;11 cells. Data are shown by the mean ± SEM of three experiments. ***P < 0.001. **b** Wright-Giemsa-stained cytospins for bone marrow samples isolated from mice engrafted with human RS4-11 cells co-transfected with the sh-NC and sh-circAF4. GFP+ cells in mice are indicated by black arrows. **c** Representative photographs of the spleen from control and RS4-11 treated mice. The spleen size and weight were notably reduced in sh-circAF4-treated mice (*n* = 3). *P < 0.05. **d** Hematoxylin and eosin staining showed the infiltration of leukemic cells in the indicated organs of mice engrafted with RS4-11 expressing the sh-circAF4 compared to control mice. GFP+ cells in tissue are indicated by black arrows. Staining was observed under a × 40 objective lens. Scar bars, 50 μm. **e** The flow cytometry result showed the substantially decreased level of GFP^+^ cells in blood and bone marrow samples form the mice treated with circAF4-knockdown RS4-11 cells versus control. These effects were accompanied by reduced blast infiltration of the spleen in circAF4-knockdown mice. **P < 0.01 and ***P < 0.001. **f** Kaplan-Meier survival curves of NOD-SCID mice transplanted with sh-NC and sh-circAF4 treated RS4-11 cells (*n* = 5 mice per group). *P* values were calculated using Log-rank (Mantel-Cox) test

We further applied a NOD-SCID mouse model [33, 37] in vivo to investigate the contribution of circAF4 in leukemogenesis. We established GFP^+^ RS4;11 cell lines which stably expressed either the circAF4 short hairpin RNA (sh-circAF4) or control short hairpin RNA (sh-NC) (Additional file 1: Figure S2a). The reduced expression of circAF4 was confirmed in sh-circAF4 cells compared with that in sh-NC cells by qRT-PCR (Fig. 3a). We injected the mice with sh-NC and sh-circAF4 RS4;11 cells by tail vein. Mice were killed after 2 weeks of injection. We found that the RS4;11 cells appeared in the bone marrow (BM) of mice (Fig. 3b), indicating the success of mice model establishment. We found that sh-circAF4 mice exhibited smaller spleens compared with the sh-NC mice (Fig. 3c), suggesting that circAF4 could regulate the infiltration of *MLL*-*AF4* leukemia. Hematoxylin and eosin (H&E) staining results also showed that the numbers of RS4;11 cells in the BM, spleen and liver from the sh-circAF4-transfected mice were reduced compared to that from the sh-NC-transfected mice (Fig. 3d). Consistently, flow cytometry analysis revealed the percentage of GFP^+^ cells was decreased in the peripheral blood, BM, spleen, liver, and lungs of the mice treated with the sh-circAF4 RS4;11 cells compared with that in the animals treated with the sh-NC-transfected cells (Fig. 3e, and Additional file 1: Figure S2b and c), suggesting that *MLL*-*AF4* leukemia may depend on circAF4 for its maintenance and progression. Notably, sh-circAF4 significantly extended survival of the mice beyond the 18 days when all of the sh-NC-treated mice succumbed to leukemia (Fig. 3f, *p* = 0.0018). Together, these data indicated that circAF4 knockdown can delay the progression of the aggressive leukemia in vivo. Notably, sh-circAF4 groups survived longer than the control groups, suggesting that circAF4, originating from the *MLL* fusion partner *AF4* gene, could regulate the infiltration of *MLL*-*AF4* leukemia.

### CircAF4 acts as a miR-128-3p sponge

**Fig. 4:**
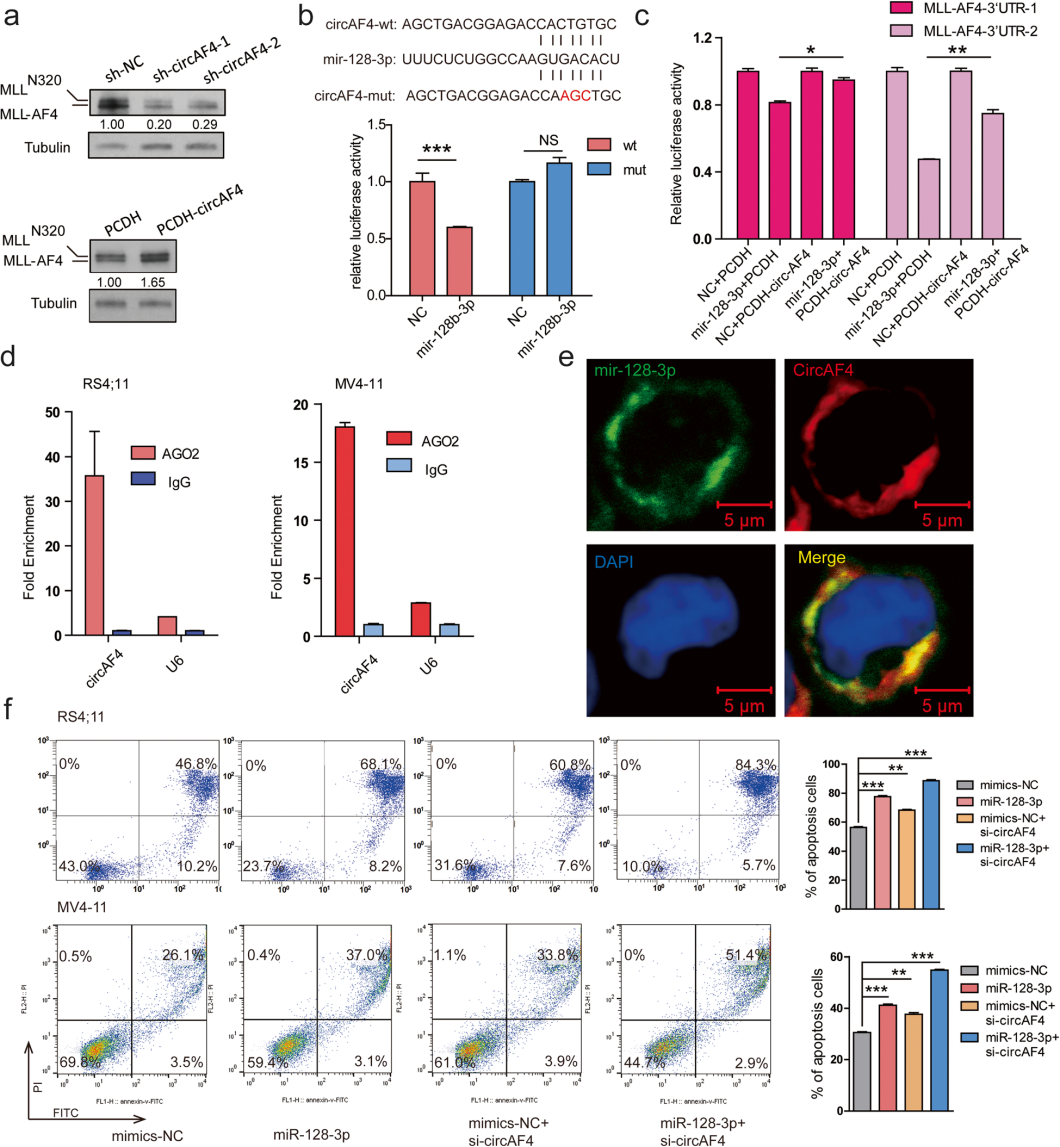
CircAF4 acts as a miR-128-3p sponge, releasing its inhibition on MLL-AF4 expression. **a** Western blotting confirmation of the effects of forced expression and knockdown of circAF4 by lentiviral constructs on MLL-AF4 protein level in RS4;11 cells. The MLL-N320 or -AF4/ Tubulin densitometric ratio was recorded by ImageJ. **b** Schematic representation of the constructs used in the luciferase assay. The sequences shown below indicate the putative miR-128-3p target site on the wild-type 3′ UTR (construct circAF4-wt), its mutated derivative (construct circAF4-mut), and the pairing regions of miR-128-3p. Luciferase assays show that transfection miR-128-3p mimic repressed the reporter activity of circAF4-wt, but not circAF4-mut. NS, not significant. ***P < 0.001. **c** Luciferase assays show that transfection miR-128-3p mimic repressed the reporter activity of MLL-AF4-3′UTR, and such repression could be partially restored by overexpression of circAF4. *P < 0.05 and **P < 0.01. **d** RNA immunoprecipitation (RIP) of AGO2 in RS4;11 and MV4-11 cells using the Magna RIP RNA-Binding Protein Immunoprecipitation Kit (Millipore, Bedford, MA) according to the manufacturer’s instructions. Analysis of precipitated RNAs: circAF4 and U6, negative control. Data are given as mean ± SEM. **e** Co-localization between miR-128-3p and circAF4 was observed by RNA in situ hybridization in RS4;11 cells. CircAF4 oligonucleotides were labeled with rhodamine (red), and miR-128-3p oligonucleotides were labeled with FAM (green). Nuclei were stained with DAPI (blue). Scar bars, 5 μm. **f** Flow cytometric analysis of Annexin V and PI positive cells in RS4;11 and MV4-11 cells transfected with indicated siRNA or miRNA mimics, respectively. *P < 0.05, **P < 0.01, and ***P < 0.001

The findings above implied that circAF4 has an oncogenic role and might be dependent on the fusion gene *MLL*-*AF4* in leukemia. Thus, we next investigated the effects of circAF4 on MLL-AF4 fusion protein via modulating circAF4 expression level. Stable knockdown of circAF4 was achieved by shRNAs as described above. Ectopic overexpression of circAF4 was achieved according to the notion that reverse complementary matches are a conserved feature of circRNA biogenesis [38]. On average, stably overexpressing circAF4 cells had 35-fold higher circAF4 expression levels compared with endogenous circAF4 (Additional file 1: Figure S3a). As shown in Fig. 4a, knockdown of circAF4 dramatically decreased the protein levels of MLL-AF4, while overexpressing circAF4 increased MLL-AF4 protein levels, showing positive association between circAF4 and MLL-AF4 fusion protein. To further confirm this finding, we performed an immunohistochemistry assay using the tissue from the sh-NC/cricAF4 RS4;11 mice. We found the decreased MLL-AF4 protein levels in the BM, spleen, and liver of the sh-cricAF4 RS4;11 mice when compared to the levels in the sh-NC RS4;11 control mice (Additional file 1: Figure S3b). These results indicated that circAF4 affected MLL-AF4 fusion protein expression level.

We next investigated the underlined mechanism that circAF4 regulates MLL-AF4 fusion proteins. It has been well documented that the subcellular location of ncRNAs can result in different functions in terms of cell fate due to their unique mechanisms [39]. Therefore, we performed qRT-PCR and fluorescence in situ hybridization (FISH) to show the cellular sublocalization of the circAF4. As shown in Additional file 1: Figure S3c and d, circAF4 was preferentially localized in the cytoplasm. Recent studies have shown that circRNAs may serve as miRNA sponges in the cytoplasm to regulate their target genes [40, 41]. Notably, previous studies revealed that the *AF4* gene is the direct target of miR-128-3p, a miRNA that acts as an anti-tumor gene via targeting MLL-AF4 [42]. MLL-AF4 3′-UTR contains two miR-128-3p binding sites. We further validated this direct targeting of miR-128-3p on the MLL-AF4 3′UTR by dual luciferase reporter assay in the study and the effect of miR-128-3p on MLL-AF4 protein level (Additional file 1: Figure S3e–h). Interestingly, by analyzing AGO2-Clip-seq data [43], miR-128-3p was found to bind to circAF4 (Additional file 1: Figure S3h). We also found that the relative expression of miR-128-3p remained almost unchanged when knocking down or overexpressing circAF4 in MV4-11 and RS4;11, suggesting that circAF4 could not regulate miR-128-3p expression (Additional file 1: Figure S3i). We thus asked whether circAF4 shared the same miRNA response elements (MRE) in the 3′-UTR of the *MLL*-*AF4* gene and functions as a ceRNA for miR-128-3p to facilitate *MLL*-*AF4* expression.

To experimentally validate this interaction, we performed luciferase assay. Luciferase reporters containing circAF4 fragment (circAF4-wt) or a construct with complementary miR-128-3p binding region mutated (circAF4-mut) were introduced into 293T cells. Reporter activity showed that approximately 40% suppression was found in the presence of circAF4-wt, but no significant suppression was shown in circAF4-mut (Fig. 4b), indicating that circAF4 can bind to miR-128-3p directly.

### CircAF4 regulates MLL-AF4 expression via releasing inhibition of miR-128-3p on the fusion protein

The results above showed circAF4 directly binds to miR-128-3p and acts as a sponge. We then investigate if circAF4 regulates *MLL*-*AF4* expression through miR-128-3p. We co-transfected the following four combinations into 293T cells: (1) MLL-AF4 3′-UTR double luciferase report vector with NC (miRNA mimics control) and empty vector PCDH, (2) MLL-AF4 3′-UTR double luciferase report vector with miR-128-3p and empty vector PCDH, (3) MLL-AF4 3′-UTR double luciferase report vector with NC and PCDH-circAF4 (circAF4 overexpression vector), and (4) MLL-AF4 3′-UTR double luciferase report vector with miR-128-3p and PCDH-circAF4. The results showed that the luciferase activity of MLL-AF4 3′-UTR was significantly inhibited by transfection of miR-128-3p compared with NC, while transfection of PCDH-circAF4 rescued the luciferase activity suppressed by miR-128-3p (Fig. 4c). The results indicated that circAF4 promoted MLL-AF4 expression through competing for binding with miR-128-3p in 293T.

Moreover, to validate this interaction, we performed RNA immunoprecipitation (RIP) of AGO2 in RS4;11 and another *MLL*-*AF4* leukemic cell, MV4-11. We observed that circAF4 was specifically bound by AGO2 (Fig. 4d). RNA FISH experiments also showed that circAF4 co-localized with miR-128-3p in the cytoplasm in RS4;11 cells (Fig. 4e). Collectively, these data indicate that circAF4 functioned as a molecular sponge of miR-128-3p to upregulate *MLL*-*AF4* expression in leukemia.

CircAF4 was demonstrated as an oncogene in *MLL*-*AF4* leukemia in the study, and miR-128-3p showed tumor suppressor function [42]. We finally asked if loss of circAF4 synergizing with miR-128-3p mimics could induce an increased rate of growth arrest and apoptosis. We forced expression of miR-128-3p and simultaneously knocked down circAF4 in RS4;11 and MV4-11 cells to detect apoptosis rate. As shown in Fig. 4f, transfection of miR-128-3p and si-circAF4, respectively, induced cell death and a higher apoptosis rate was observed when co-transfection of miR-128-3p and si-circAF4. These data clearly show that circAF4, generated from its parent gene *AF4*, regulates the expression level of *MLL*-*AF4* by directly targeting miR-128-3p and then promotes leukemia progression. Targeting circAF4 together with miR-128-3p mimics could be a potential strategy for treating the disease.

**Fig. 5:**
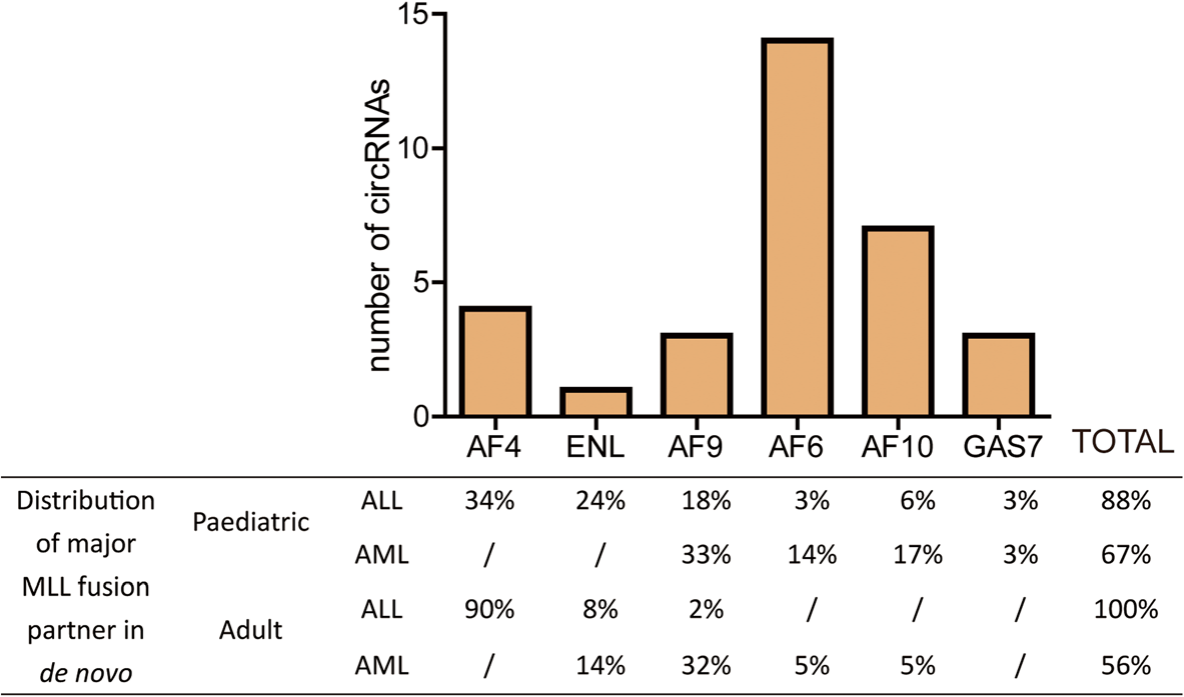
Search for the FP-circRNA. The number of circRNAs derived from *MLL* fusion partner gene according to a large circRNA profiling study. The distribution of these major *MLL* fusion partner in de novo was showed in the table below

We finally asked whether any other circRNAs could originate from partner genes during MLL translocation. We then examined six other MLL fusion partner genes (*AF4*, *ENL*, *AF9*, *AF6*, *AF10*, and *GAS7*) in the same circular RNA profiling study [29]. The results showed that a number of circRNAs could indeed originate from these partner genes (Fig. 5). Further studies are necessary to demonstrate their function in the disease progression.

## Discussion

Chromosome rearrangements involving 11q23, fusing *MLL* gene to various fusion partners, can result in leukemia with poor prognosis and *MLL*-*AF4* is the most frequent *MLL* rearrangements. A better understanding of the molecular mechanisms underlying the pathogenesis of *MLL*-*AF4* leukemia could improve disease treatment. In this study, we found a new regulatory way for *MLL*-*AF4* post-transcriptional regulation via specific circRNA-parental mRNA-miRNA axes. We show for the first time that circAF4 plays an oncogenic role in *MLL*-*AF4* leukemia. CircAF4 originates from the *MLL* fusion gene partner *AF4* gene. It can act as a miR-128-3p sponge, thereby releasing its inhibition on *MLL*-*AF4* expression. Thus, targeting circAF4 can reduce *MLL*-*AF4* expression and induce the apoptosis of leukemia cells. More importantly, we found that knockdown of circAF4 and overexpression of miR-128-3p may have cooperative effects in inducing apoptosis; therefore, the combination of these two molecular mechanisms may be a promising target for disease therapy. Thus, our study reveals a previously unrecognized mechanism of *MLL*-*AF4* gene regulation.

Fusion circRNAs (f-circRNAs) have been found to increase the cellular proliferation rate and provide tumor cells a survival advantage upon therapy treatment both in vitro and in vivo [5], but only a small set of f-circRNAs could be generated from the fusion regions. An example is only f-circM9 to be produced from the fusion region of *MLL*-*AF9* gene in leukemia; however, in *MLL*-rearranged leukemia, the *MLL* gene can be fused with one of over 100 partners, including *AF4*, *AF9*, and *ENL*, to form more than 100 *MLL* fusion genes [28]. Therefore, we analyzed six of the most frequent *MLL* rearrangements including *MLL*-*AF4*, *MLL*-*ENL*, *MLL*-*AF9*, *MLL*-*AF6*, *MLL*-*AF10*, and *MLL*-*GAS7*, which account for approximately 60% of all *MLL* translocation-bearing leukemia [31]. It was shown that a number of circRNAs could originate from these partners, suggesting the potential of fusion gene partner-derived circRNAs (we named these kind of circRNAs as FP-circRNAs) as therapeutic targets for leukemia with chromosomal translocations. Future studies are necessary to address the other FP-circRNAs from different fusion partners during gene translocation, and whether the formed circRNAs have an effect on fusion genes and cancer cell activities.

## Conclusion

In summary, we demonstrated that the abnormal elevated expression of circAF4 is correlated with *MLL*-*AF4* leukemia. CircAF4 promoted *MLL*-*AF4* leukemia cell growth by acting as a molecular sponge of miR-128-3p to regulate MLL-AF4 expression. These results suggest that circAF4 may be a novel therapeutic target of *MLL*-*AF4* leukemia.

## Supplementary information

**Additional file 1: Figure S1.** The association of circAF4 expression of the clinical pathological data characteristics and function in leukemia. **Figure S2.** CircAF4 regulates the *MLL* leukemia progression in vivo. **Figure S3.** CircAF4 regulates the *MLL*-*AF4* expression by binding to miR-128-3p in a ceRNA manner.

## Data Availability

The material supporting the conclusion of this study has been included within the article.

## Abbreviations

*AF4*: AF4/FMR2 family member 1
*AF9*: MLLT3 super elongation complex subunit
AGO2: Argonaute RISC catalytic component 2
ALL: Acute lymphoblastic leukemias
BM: Bone marrow
CCK8: Cell Counting Kit
ceRNAs: Competing endogenous RNAs
circRNAs: Circular RNAs
CR: Complete remission
f-circRNA: Fusion circRNA
FISH: Fluorescence in situ hybridization
FP-circRNAs: Fusion gene partner-originating circRNAs
H&E: Hematoxylin and eosin
miRNAs: MicroRNAs
*MLL*: Mixed-lineage leukemia
ncRNA: Noncoding RNAs
qRT-PCR: Quantitative reverse transcription-PCR
RIP: RNA-Binding Protein Immunoprecipitation
shRNA: Short hairpin RNA
siRNAs: Small interfering RNAs
