# Supplementary material for: circRNA circAF4 functions as an oncogene to regulate MLL-AF4 fusion protein expression and inhibit MLL leukemia progression
Source: J Hematol Oncol. 2019 Oct 17;12:103. doi: 10.1186/s13045-019-0800-z (PMC6798510; doi:10.1186/s13045-019-0800-z)
Supplement: Supplementary file 1 — Additional file 1: Figure S1. The association of circAF4 expression of the clinical pathological data characteristics and function in leukemia. Figure S2. CircAF4 regulates the MLL leukemia progression in vivo. Figure S3. CircAF4 regulates the MLL-AF4 expression by binding to miR-128-3p in a ceRNA manner. [file 13045_2019_800_MOESM1_ESM.docx]

**Additional file 1 for “CircRNA CircAF4 Functions as An Oncogene to Regulate MLL-AF4 Fusion Protein Expression and Inhibit *MLL* Leukemia Progression”**


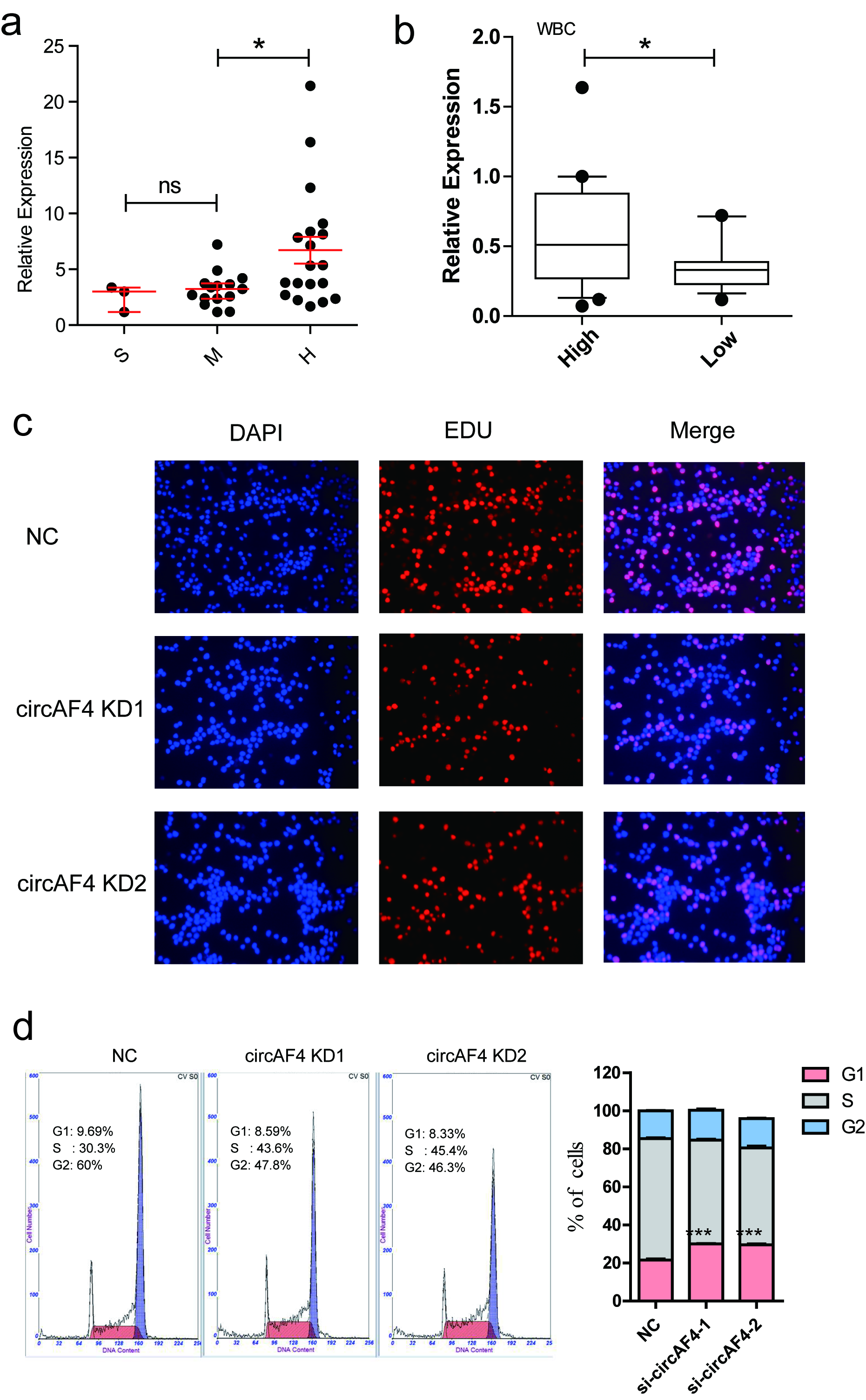


**Figure S1** The association of circAF4 expression of the clinical pathological data characteristics and function in leukemia. **a**, the patients were clustering into three risk stratification groups, standard risk (S), middle risk (M) and high risk (H), based on the risk stratification system from ALLIC BFM 2002, and found the patients in the high risk group had a highest circAF4 expression. NS: no significant; *, P<0.05. **b**, patients who have higher white blood cells (WBC) may express higher level of circAF4. High: the patients with the top half of WBC; Low: the patients with the bottom half of WBC; *, P<0.05. **c** The cell proliferation was also tested by EdU assay using Cell-Light EdU Kit (RiboBio, China). RS4;11 cells were seeded in 48 wells, 48 hours after electrotransfected with si-circAF4 or negative control (NC) oligonucleotide. Cells were added with 50 mM EdU and incubated for another 4 h. Cells were then fixed with 4% paraformaldehyde and stained with Apollo Dye Solution for proliferating cells. Nucleic acids in all cells were stained with Hoechst 33342. Images were taken using a fluorescence microscope. **d** Flow cytometry was performed to detect the effects of circAF4 on cell cycle. For cell cycle analysis, transfected cells were harvested and washed twice with cold PBS, and the Cell Cycle Kit (MULTISCIENCES, Hangzhou, China) was used according to the manufacturer’s guidelines. The detection was performed with a FACS Calibur using CellQuest software (BDIS, San Jose, CA, USA).


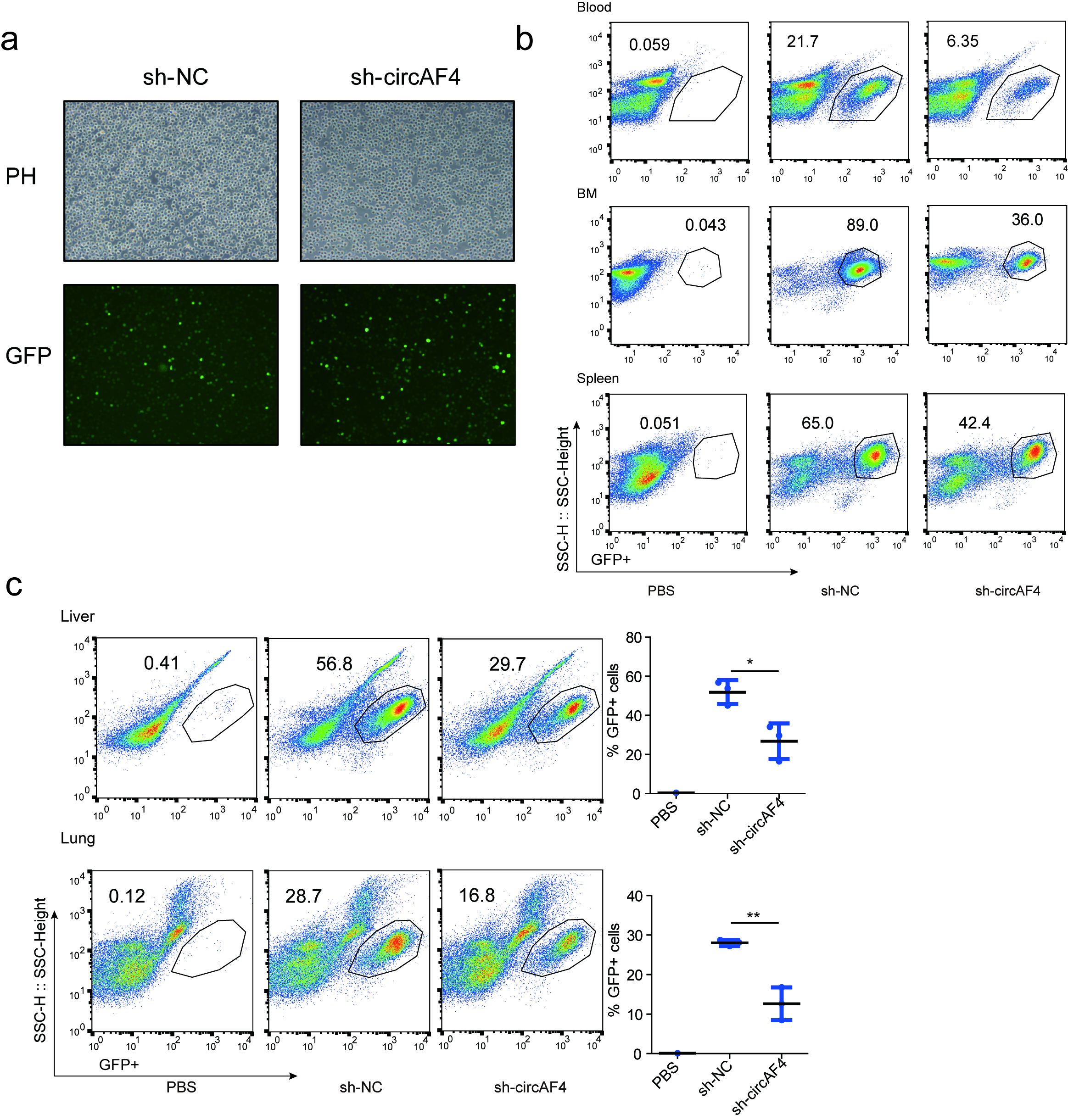


**Figure S2** CircAF4 regulates the *MLL* leukemia progression *in vivo*. **a** Construction of GFP^+^ sh-NC and GFP^+^ sh-circAF4 RS4;11 cells. The flow cytometry result showed the substantially decreased level of blasts in blood, BM, spleen **(b)**, liver and lung **(c)** samples form the mice treated with circAF4-knockdown RS4-11 cells versus control. These effects were accompanied by reduced blast infiltration of the liver and spleen in circAF4-knockdown mice. *P<0.05, **P<0.01 and ***P<0.001.


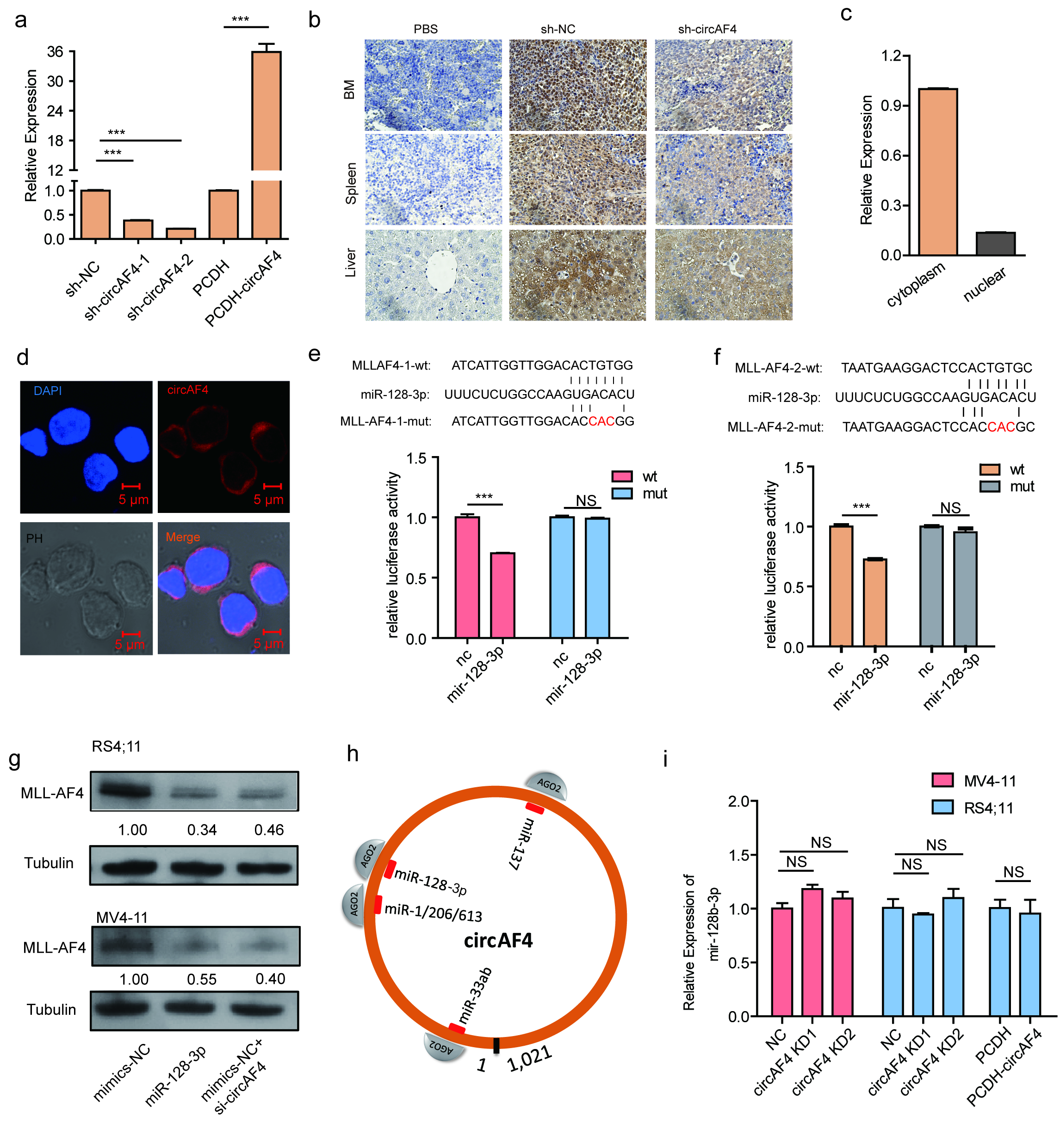


**Figure S3** CircAF4 regulates the *MLL-AF4* expression by binding to miR-128-3p in a ceRNA manner. **a** QRT-PCR confirmation of knockdown and overexpression of circAF4 by lentiviral constructs in RS4;11 cells. Data are the means±s.e.m. of three experiments. ***, P<0.001. **b** Immunstaining for MLL-AF4 in BM, spleen and liver samples form the mice treated with circAF4-knockdown RS4-11 cells versus control. **c** QRT–PCR data indicating the abundance of circAF4 in either the cytoplasm or nucleus of RS4;11 cells. The nuclear and cytoplasmic fractions were extracted using NE-PER Nuclear and Cytoplasmic Extraction Reagents (Thermo Scientific, USA). Total RNA from whole-cell lysates or the nuclear and cytoplasmic fractions were isolated using TRIzol (Life Technologies, USA). Western blot analysis of H3 and Actin is shown on the right. **d** RNA fluorescence *in situ* hybridization for circAF4. CircAF4 oligonucleotides were labeled with rhodamine (red) and nuclei were stained with DAPI (blue). Scar bars, 5μm. **e-f** Schematic representation of the constructs used in the luciferase assay. The sequences shown below indicate the putative miR-128-3p target site on the wild-type 3’ UTR (construct MLL-AF4-wt), its mutated derivative (construct MLL-AF4-mut), and the pairing regions of miR-128-3p. 293T cells were co-transfected with pSi-Check2 with either circAF4-wt or circAF4-mut and with miR-128-3p or NC. Each Renilla luciferase reading was normalised to that obtained for the control firefly luciferase. NS: no significant; ***, P<0.001. **g** Western blotting of MLL-AF4 protein level under knockdown of circAF4 or transfection of miR-128-3p mimics in RS4;11 cells. The MLL-AF4/ Tubulin densitometric ratio was recorded by ImageJ. **h** A schematic drawing showing the putative binding sites of the miRNAs associated with circAF4 in AGO2 clip-seq analysis. **i** QPCR of miR-128-3p under knockdown or overexpression of circAF4 in MV4-11 and RS4; 11. NS: no significant.
